# Assessing parallel gene histories in viral genomes

**DOI:** 10.1186/s12862-016-0605-4

**Published:** 2016-02-05

**Authors:** Beatriz Mengual-Chuliá, Stéphanie Bedhomme, Guillaume Lafforgue, Santiago F. Elena, Ignacio G. Bravo

**Affiliations:** Infections and Cancer Laboratory, Catalan Institute of Oncology (ICO), Barcelona, Spain; Bellvitge Institute of Biomedical Research (IDIBELL), Barcelona, Spain; Centre d’Ecologie Fonctionnelle et Evolutive, UMR CNRS 5175, Montpellier, France; Instituto de Biología Molecular y Celular de Plantas, Consejo Superior de Investigaciones Científicas-Universidad Politécnica de Valencia, València, Spain; I2SysBio, Consejo Superior de Investigaciones Científicas-Universitat de València, València, Spain; The Santa Fe Institute, Santa Fe, NM USA; MIVEGEC (UMR CNRS 5290, IRD 224, UM), National Center for Scientific Research (CNRS), Montpellier, France; National Center for Scientific Research (CNRS), Maladies Infectieuses et Vecteurs: Ecologie, Génétique, Evolution et Contrôle (MIVEGEC), UMR CNRS 5290, IRD 224, UM, 911 Avenue Agropolis, BP 64501, 34394 Montpellier, Cedex 5 France

**Keywords:** Gene trees, Incongruence, Phylogenetic inference, Species trees, Virus evolution, Pathogen evolution, Potyvirus, Papillomavirus, HPV

## Abstract

**Background:**

The increasing abundance of sequence data has exacerbated a long known problem: gene trees and species trees for the same terminal taxa are often incongruent. Indeed, genes within a genome have not all followed the same evolutionary path due to events such as incomplete lineage sorting, horizontal gene transfer, gene duplication and deletion, or recombination. Considering conflicts between gene trees as an obstacle, numerous methods have been developed to deal with these incongruences and to reconstruct consensus evolutionary histories of species despite the heterogeneity in the history of their genes. However, inconsistencies can also be seen as a source of information about the specific evolutionary processes that have shaped genomes.

**Results:**

The goal of the approach here proposed is to exploit this conflicting information: we have compiled eleven variables describing phylogenetic relationships and evolutionary pressures and submitted them to dimensionality reduction techniques to identify genes with similar evolutionary histories. To illustrate the applicability of the method, we have chosen two viral datasets, namely papillomaviruses and *Turnip mosaic virus* (TuMV) isolates, largely dissimilar in genome, evolutionary distance and biology. Our method pinpoints viral genes with common evolutionary patterns. In the case of papillomaviruses, gene clusters match well our knowledge on viral biology and life cycle, illustrating the potential of our approach. For the less known TuMV, our results trigger new hypotheses about viral evolution and gene interaction.

**Conclusions:**

The approach here presented allows turning phylogenetic inconsistencies into evolutionary information, detecting gene assemblies with similar histories, and could be a powerful tool for comparative pathogenomics.

**Electronic supplementary material:**

The online version of this article (doi:10.1186/s12862-016-0605-4) contains supplementary material, which is available to authorized users.

## Background

One of the key goals of evolutionary biology is to reconstitute the evolutionary history of species and to establish their filiation patterns. This goal has been pursued using first morphological and physiological data and later molecular data, which harbour a large amount of phylogenetic information. The first molecular phylogenetic reconstructions were based on, often partial, sequences of one orthologous locus in various species. A single locus was considered representative of the history of the whole genome and of the species. As more sequence data became available, this representativeness was jeopardized as examples of incongruent stories revealed by different genes for the same set of species or discrepancies between species tree and gene trees accumulated, e.g. fungi [1, 2], plants [3] and mammals [4]. Earlier examples have been reviewed by Nichols and coworkers [5]. The reason for these discrepancies can be either biological or technical. Regarding biology, there are three main evolutionary events responsible for them [6]. The first one is incomplete lineage sorting, also called deep coalescence, which corresponds to the persistence, after speciation, of ancestral polymorphism and subsequent loss of alleles or random sampling. For example incomplete lineage sorting has been pervasive during the bird adaptive radiation that followed the Cretaceous/Tertiary crisis [7]. The second one is gene exchange between species, which can occur either by hybridization, pervasive in plants [8], or by horizontal gene transfer, frequent in prokaryotes [9, 10]. The third one is gene duplication and subsequent loss and evolution that might render difficult the correct identification of orthologous genes [11]. Regarding reconstruction techniques, discrepancies between gene tree and species tree inferences can also be artefacts due among others to sequencing errors, orthologous genes misidentification, alignment underperformance, wrong model choice or inefficient search for global likelihood optima during phylogenetic reconstruction.

In the genomic era, datasets span several genes (sometimes the whole genome), each available in a variable number of taxa. A higher volume of sequences means more phylogenetic information but also more incongruences between gene trees. This renders the reconstitution of species trees always more difficult and controversial. The two main questions about incongruences are: (1) how to detect and quantify them? and (2) what to do with them? Regarding the first question, a large number of tests have been designed to compare two phylogenetic trees, assessing either the distance or similarity in terms of topology or branch length or a combination of both. An idea of the diversity of these methods as well as a test for their relative efficiency depending on the dataset can be found in Kuhner and Yamato [12]. Additionally, methods have been developed to analyse sets of phylogenetic trees reconstructed from different sequences. They allow identifying outliers, that can be futher studied to determine the origin of the difference in their reconstructed evolutionary history. These methods use principal components analysis [13], heat maps [14] or clustering of likelihood ratio tests [15], Euclidean distances [16], multiple co-inertia analysis [17], linear correlation between genetic distance matrices of aligned individual gene sequences and aligned genome sequences [18], information theory [19], or non-parametric estimation of tree distribution [20].

Regarding the “what to do with them?” question, one approach has been to find a consensus tree capturing the essential features of the evolutionary history of the species. Data for each gene can be analysed independently and then combined by a consensus tree approach [21, 22], supertree-based approaches [23–26], Bayesian approach [27], summary by maximum agreement subtrees [28, 29], coalescent approach (e.g. [30]), or Bayesian reconstruction of gene trees taking the species tree as a prior [31, 32]. An extension of this last model proposes the estimation of the species tree from multiple-allele data [33]. Data for each gene can also be analysed simultaneously through concatenation into a supermatrix [34]. Recent developments allow for differential weighing between partitions, e.g. as a function of parameters such as gene length or bootstrap support, in order to avoid arbitrarily giving the same importance to all genes and all partitions, as synthetized by de Queiroz and coworkers [35]. Conflict between gene trees can also be seen as a source of information about genome evolution rather than an obstacle to reconstructing the species tree. Some methods acknowledge that a genome contains different evolutionary histories, either through gene networks representing alternative phylogenetic paths in a graphical way or the projection of conflicting signals in 2D, (e.g. SplitsTree4; [36]), or by explicit consensus network, in which horizontal gene transfers and hybridization are explicitely represented [37]. Additionally, methods have been developed that extract several consensus trees from a set of gene trees, by clustering output trees [38], by representing all splits above a predefined threshold in as few trees as possible [39] or by introducing a general score that compares the goodness of fit of models with one or various trees [40].

The available methods generally focus on a single characteristic extracted from each gene alignment, most often, the topology of the best-known tree. Some methods can be applied to more than one characteristic, for example the nodal and the patristic distance, but none of them allows combining various evolutionary characteristics and extracting in a synthetic way groups of genes with similar evolutionary history. Recent methods have been developed to perform analysis on very large data sets and often do not perform well on small genomes. For example, using simulated data sets, de Vienne and coworkers [17] established that Phylo-MCOA retrieved correctly outliers only if data sets contained more than 30 genes and species. Thus, such methods may not be suited for application to small viral genomes, in which each gene represents a significant proportion of the whole genome.

Here we propose a method to identify groups of genes sharing similar evolutionary histories using an integrative strategy. In this strategy, we have considered different characteristics of the evolution of each of the genes, such as tree-topology, branch length, detection of the level of selection operating on the proteins and phylogenetic distances between taxa, and have combined them in order to detect groups of genes sharing evolutionary characteristics. We applied this method to viral data sets. The small size of viral genomes is not synonymous of simple and homogeneous evolutionary history. Indeed, viral evolution is strongly affected by recombination and by differential selection pressure on different genes. In vivo estimates of recombination rate range from 4 × 10^-8^ in *Poliovirus* [41] to 10^-4^ in *Hepatitis C virus* [42]. Recombination has been documented to occur at the intraspecific level as in *Watermelon mosaic virus* [43], at the intragenus level as in *Potyvirus* [44], at the intrafamily level as between *Ipomovirus* and *Potyvirus* [45] or between *Papillomavirus* genera [46] and at the interfamily level as between *Papillomaviridae* and *Poliomaviridae* [47]. Besides, differential selection pressures acting on different genes result in diverse evolutionary rates, for example in *Hepatitis E virus* [48], in porcine parvoviruses [49] or in murine cytomegalovirus [50]. Typical genes with a high proportion of codons under positive selection are the ones in contact with the host immune system and implicated in immune escape and evolutionary arms race [51, 52].

## Methods

**Fig. 1:**
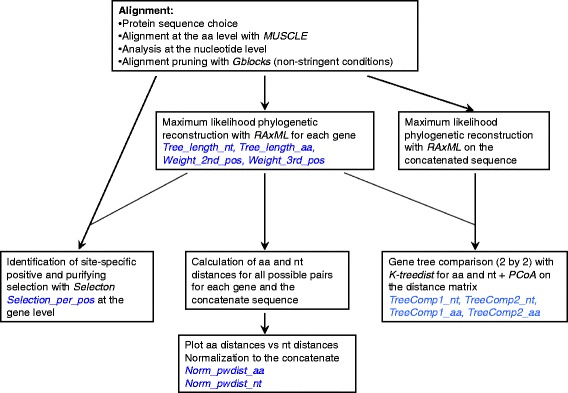
Diagram representing the different evolutionary parameters (in red) derived from the initial protein sequence data set of the different genes and their concatenated. These eleven parameters are those included in the final cluster/PCA

The general analysis strategy is depicted in Fig. 1 and a detailed workflow description is depicted in Additional file 1: Supplementary material and methods. We have collected information about the gene-specific evolutionary patterns at three levels: direct phylogenetic inference, selection, and pairwise evolutionary distances.

### Phylogenetic inference

Viral gene sequences were aligned at the amino acid level using Muscle [53] and the underlying nucleotide sequences were aligned accordingly. Poorly aligned sequences and divergent regions were removed using the Gblocks software [54] at the codon level under non-stringent conditions and allowing for gap positions. Phylogenetic relationships were inferred separately for each gene and for the concatenated sequence of all genes in a genome. Phylogenetic reconstructions were performed by maximum likelihood (ML) using RAxML_v7.2.8 [55] at the nucleotide level, using the GTR + G4 model, with partitions per gene and per codon position within a gene, and 500 bootstrap cycles, and at amino acid level using the LG + G4 model. Model choice was performed using the Akaike information criterion among alternative models tested with RAxML for nucleotide and for amino acids. To visualize and represent incongruences between gene trees, we generated a split network computed from gene trees as a supernetwork using 100 runs of the SplitsTree4 software with default parameters [36]. Phylogenetic reconstructions provided the first four variables for the analysis: total tree length for the nucleotide tree (*Tree_length_nt*), relative contribution of the second and third codon positions to the tree length (*Weight_2nd_pos*, *Weight_3rd_pos*), and total tree length for the amino acid tree (*Tree_length_aa*).

### Tree comparison

Evaluating similarities between phylogenetic trees is still a source of debate, and several methods for tree comparison have been proposed, possibly because there is no simple answer to the description of the extent of difference between trees, as reviewed recently [12]. To quantify the overall differences in the relative branch length and topology of two phylogenetic trees, we have chosen the *K*-score index, which searches to minimise the branch length distance between both trees [56]. The method is implemented in the Ktreedist software. We calculated all pairwise K-score values between gene trees at the amino acid and at the nucleotide levels. Importantly, the K-score calculation involves first a scaling step that is dependent on the tree used as reference, and the resulting matrices are therefore asymmetrical. To obtain values describing the general characteristic of one gene in terms of similitude of its tree to the trees of the other genes, these matrices were processed using Principal Coordinate Analysis (PCoA) with the vegan and ade4 R Packages. The output of the PCoA provides a re-scaled distance matrix between the phylogenetic trees used as input, with the new, re-scaled dimensions accounting for a decreasing amount of the overall variability in the initial matrix. In our case, the first two dimensions of the PCoA, captured a large proportion of the variance: above 60 % for TuMV and above 90 % for PVs. Both dimensions were plotted in order to visualize close vs. distant genes in terms of similitude of their trees. The coordinates of each gene on the first two dimensions were retained for the final analyses (*TreeComp1_nt*, *TreeComp2_nt, TreeComp1_aa, TreeComp2_aa*).

### Selection regime

For each gene, signature of positive, negative selection or lack thereof was identified by calculating the *ω* = *d*_*N*_/*d*_*S*_ ratio at the individual codon level. For each gene, the alignment and the best-known ML tree were used as input for the Selecton online tool [57]. We assessed first the presence of positions under positive selection using the MEC [58] and the M8 evolutionary models, and tested likelihood against the alternative M8a model, which does not consider positive selection [59]. The Huber robust central M-estimator of *ω* was used as a synthetic value of the selection direction for each gene and saved as an additional evolutionary parameter (*Selection_per_pos*). In order to have high power under normality the constant used to calculate the Huber M-estimator was 1.28.

### Pairwise evolutionary distances

**Fig. 2:**
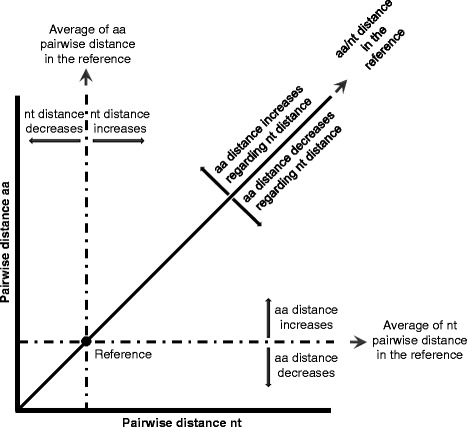
Normalised nucleotide distance *vs* normalised amino acid distance. The (1, 1) point labels the evolutionary behaviour of the concatenated and the lines parallel to the axis and passing through this point define four sectors representing the four types of relative evolutionary speed

For each gene tree and for the corresponding concatenated tree, all pairwise patristic distances (*pwdist,* i.e., the sum of branch-length distances) between terminal taxa were calculated both at the amino acid and nucleotide levels. In order to compare distances between taxa for different genes in a same genome, we normalised them by dividing each pairwise distance obtained from a gene tree by the corresponding distance obtained from the concatenated tree for the same pair of taxa. For each gene, normalised distances were synthesized by calculating the Huber robust central M-estimator and the associated median absolute deviation (MAD). The Huber M-estimator of pairwise nucleotide distances and amino acid distances were added as evolutionary parameters characterizing the genes (*Norm_pwdist_nt*, *Norm_pwdist_aa*). Additionally, the graphical representation shown in Fig. 2 of the normalised amino acid distance versus the normalised nucleotide distance allows categorizing the genes for their evolutionary behaviour relatively to the genome containing them. Indeed, the (1,1) point represents the evolutionary characteristics of the concatenated, and lines parallel to the axes and going through this point divide the plan in four sectors of faster/slower evolution for amino acid/nucleotide (Fig. 2).

Finally all the above-mentioned evolutionary parameters (shown in blue in Fig. 1) were combined to identify genes displaying similar evolutionary metrics by applying techniques for dimensionality reduction implemented in the R packages pvclust and stats: i) Ward hierarchical cluster identification using the Euclidean distances. The approximately unbiased (AU) support values, and the bootstrap probability (BP) support values were also computed; ii) principal component analysis (PCA). Data were standardized prior to their analysis.

### Data sets

#### *Turnip mosaic virus* (TuMV) data set

The first data set used contained 30 genomes of TuMV isolates (Additional file 2: Table S1a). TuMV belongs to the genus *Potyvirus* within the family *Potyviridae*, the largest family of plant viruses and the source of important crop losses in cultivated plants. TuMV is a worldwide distributed virus, sap-transmissible to a wide range of species and transmitted by many aphid species in a non-persistent manner [60]. TuMV is a positive-sense single stranded RNA virus, with a filamentous, non-enveloped helical capsid. As all potyviruses, the TuMV genome is linear, monopartite and around 10 kb in length. It encodes a unique ORF, translated into a polyprotein, autocatalytically cleaved into ten mature proteins: P1, HC-Pro, P3, 6 K1, CI, 6 K2, VPg, NIa-Pro, NIb, and CP. An overlapping open reading frame coding a small additional protein, PIPO, after +2 frameshifting within P3 has also been described [61]. The knowledge of the intra-plant biology of this virus is moderate, compared to viruses infecting humans, and the protein function(s) are well described for some proteins, yet ignored for others. There are currently over one hundred whole TuMV genome sequences available but a minimum recombination set of 30 TuMV full-length genomes were retained for the analysis, according to Tan and coworkers [62]. Only coding sequences were used for this analysis, and the *PIPO* overlapping gene was not considered.

#### *Papillomavirus* data set

The second data set contained genomes of various papillomavirus species (Additional file 2: Table S1b). Papillomaviruses (PVs) are animal viruses belonging to the *Papillomaviridae* family. The biology and natural history for some of these viruses and the function of each gene are known in many details, because of their role in inducing lesions, benign and malignant tumours in humans and animals [63]. PVs are small, non-enveloped viruses, with a genome encoded in a circular double-stranded DNA molecule of around 8 kb. The PV genome is divided in two gene clusters. The first one called “early genes” is composed by E6 and E7 (involved in the initial destabilization of the host cell), E1 and E2 (genome replication), E4 (interaction with the cellular cytoskeleton), and E5 (immune exposure and response to growth factors) [64]. The second cluster called “late genes” is composed by L1 and L2, encoding for the capsid proteins. The E4 ORF, nested within the E2 gene, was not included in our analyses. The E5 was also not considered in our analyses because it is absent in most PV genomes, and probably they do not share a common ancestor [65]. Most of the complete PV genomes deposited in databases are human PVs belonging to the *Alpha*-, *Beta*- and *Gammapapillomaviruses*. In contrast, animal PV diversity is poorly sampled. To avoid an over-representation of these three taxa, a subset of 79 representative PV types that covered the sequence diversity of all known PVs was chosen. Representative sequences were chosen avoiding PVs suspected of having undergone recombination [46].

## Results

### Analysis of the TuMV data set

Additional file 3: Figure S1a displays the best-known ML tree obtained for the concatenated sequences and Additional file 3: Figure S1b the split network resulting from the individual best-known ML gene trees. Individual gene best-known ML trees are given in Additional file 3: Figure S1 and support values for each group is shown in Additional file 4: Table S2a.

**Fig. 3:**
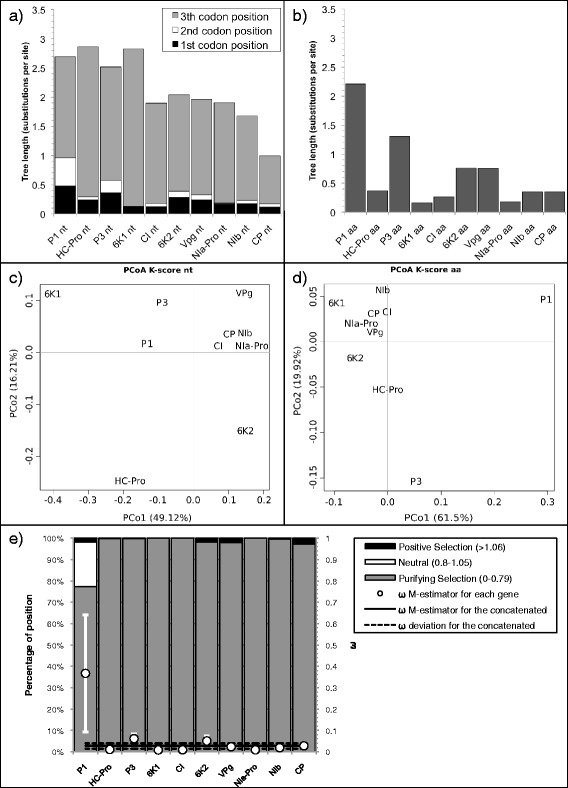
**a,b** Tree length (number of substitution per site) of TuMV. **a** Plot of the nucleotide tree length displaying the contribution of the first, second and third codon position in dark grey, white and light grey respectively. **b** Plot of the amino acid tree length. **c,d** Biplot of the principal coordinates analysis (PCoA) using the four variables *TreeComp1_nt, TreeComp2_nt, TreeComp1_aa, TreeComp2_aa* (see Additional file 5: Table S3) for TuMV. The first principal component is represented in the x-axis, and the second principal component is represented in the y-axis. Percentage values in the axes indicate the percentage of variation explained by either component. **e** Percentage of sites under positive (dark grey), neutral (white) and purifing selection (light grey) for TuMV (left scale). The solid black line represents the Huber M-estimator of *ω* (± median absolute deviation) of all positions for each gene and for the concatenated (right scale)

The nucleotide gene tree lengths (without partition) showed a decreasing trend along the TuMV genome (Fig. 3a), as indicated by the negative regression of the nucleotide tree length on the gene order (*R*^2^ = 0.82; *F*_1,8_ = 36.54, *p* < 0.001). Grouping the tree lengths by a *K*-means cluster and choosing the cluster number by the AIC, revealed that the data best cluster in three groups: the four genes located on 5’ in the genome (P1, HC-Pro, P3, and 6 K1; tree length between 2.5 and 2.9 accepted substitutions per site), the following five genes (CI, 6 K2, VPg, NIa-Pro, and NIb; tree length between 1.65 to 2) and CP (tree length 0.994). Interestingly, the amino acid gene tree length (Fig. 3b), follows the same decreasing trend but with a lot more variations resulting in a non-significant regression of tree length on gene order and no clear clustering of the tree length values (*R*^2^ = 0.33; *F*_1,8_ = 3.95, *p* =0.082). As expected, the third codon position provides the highest contribution to the total tree length, followed by the first and then the second position, the latest being fixed for 6 K1 and NIa-Pro.

Four columns of Additional file 5: Table S3a show the projection values in the first two dimensions of the PCoA of the pairwise distances between the individual gene trees, considering a combination of topology and branch length (*K*-score) (Fig. 3c and d) [56]. In terms of selection regime, all TuMV genes present a *ω* central estimator below 0.07 except P1, with a *ω* central estimator of 0.37 (Fig. 3e). Accordingly, P1 is also the gene with the highest proportion of codons under positive or neutral selection (23 %). The rest of the genes present a large majority (>95 %) of their codons under purifying selection.

**Fig. 4:**
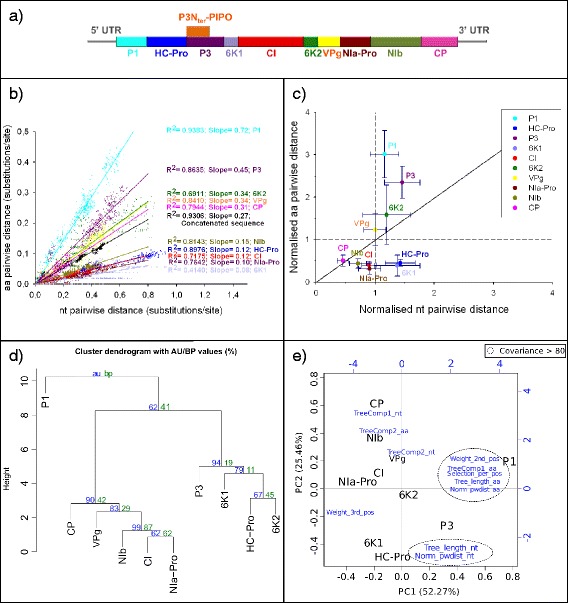
**a** TuMV genome organisation. **b** Nucleotide distance *vs* amino acid distance of the TuMV. Slopes values for each regression are indicated. All regression *p* < 0.001. **c** Nucleotide *vs* amino acid pairwise distances for TuMV genes. For each gene, the Huber estimator (±median absolute deviation) of the distances normalised to their respective concatenated is displayed. **d** Hierarchical cluster dendogram using the eleven chosen variables (see Additional file 5: Table S3a) for TuMV. The clustering was performed using the euclidean distances and the Ward method. Probability values were calculated using bootstrap resampling techniques, the approximately unbiased (AU) support *p*-value (red) and the bootstrap probability (BP) value (red). **e** Biplot of the principal component analysis (PCA) using the eleven chosen variables (see Additional file 5: Table S3a) for TuMV. The first principal component is represented in the x-axis, and the second principal component is represented in the y-axis. Percentage values in the axes indicate the percentage of variation explained by either components. Original variables are given in blue, and those showing co-variation above 0.8 are encircled by discontinuous lines

The organisation of the genes into the TuMV genome is shown in Fig. 4a. Plotting the nucleotide *pwdist* against the amino acid *pwdist* yielded always a high correlation, for each individual gene as well as for the concatenated, but the slope of the regression varied largely depending on the gene (see example in Fig. 4b). When normalising the distances by dividing each individual gene-based *pwdist* by the corresponding concatenated-based *pwdist*, different genes behaved differently and fell in three sectors of the normalised plot (Fig. 4c): P1, P3 and 6 K2 displayed both higher amino acid and nucleotide *pwdist* than the concatenated; VPg displayed higher amino acid but similar *pwdist* than the concatenated; 6 K1 and HC-Pro displayed lower amino acid but higher nucleotide *pwdist* than the concatenated; CI, NIa-Pro and NIb displayed both lower amino acid and nucleotide *pwdist* than the concatenated; and CP displayed less variation in both amino acid and nucleotide than the average of the genome, but the ratio between both was similar to that of the concatenated.

The results for the eleven variables extracted (Additional file 5: Table S3a) were finally combined and a cluster analysis was applied to the ten genes in the TuMV genome. The results are displayed in Fig. 4d. The height of the branches represents the distances between clusters calculated by the Ward method. The cluster analysis showed a clear separation of P1 from the rest of genes (AU support *p* = 0.63). Two further clusters could be distinguished, a first one containing genes located in the first half of the polyprotein (HC-Pro, P3 and 6 K1) and 6 K2, and a second one containing genes of the second half of the polyprotein and CI. Members of the second cluster were more similar between them than the one of the first one and there is no further grouping within each of these two clusters. Regarding the PCA, the first two axes explain more than 75 % of the global variance, with P1 differing largely from the rest of the genes based on the first derived axis (Fig. 4e). The value on the second axis seems to globally correspond to gene order in the genome: genes from the first half of the genome have negative values whereas genes from the second half have positive values. Covariance values above 80 cluster the eleven considered variables into two groups and four isolated variables.

### Analysis of the PV data set

Additional file 6: Figure S2a displays the best-known ML tree obtained for the concatenated genes and Additional file 6: Figure S2b the split network from the individual best-known ML gene trees. Individual gene trees are depicted in the same Additional file 6: Figure S2 and support values for the crown groups are provided in Additional file 4: Table S2b.

**Fig. 5:**
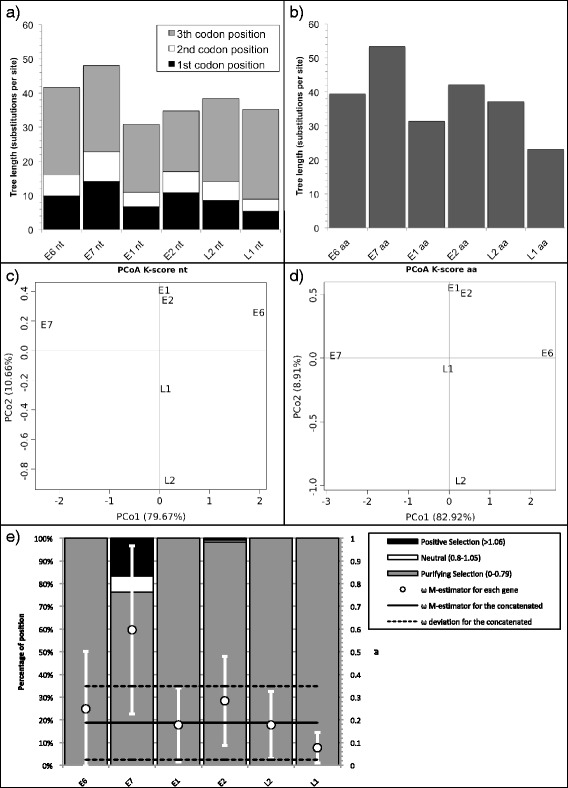
**a,b** Tree length (number of substitution per site) for PVs. **a** Displays nucleotide tree length with the contribution of the first, second and third codon position in dark grey, white and light grey respectively. **b** Represents the amino acid tree length. **c,d** Biplot of the principal coordinates analysis (PCoA) using the four variables *TreeComp1_nt, TreeComp2_nt, TreeComp1_aa, TreeComp2_aa* (see Additional file 5: Table S3) for PV. The first principal component is represented in the x-axis, and the second principal component is represented in the y-axis. Percentage values in the axes show the percentage of variation explained by either components. **e** Percentage of sites under positive (dark grey), neutral (white) and purifing selection (light grey) for PVs (left scale). The solid black line represents the Huber M-estimation of *ω* (±median absolute deviation) of all position for each gene and the concatenated (right scale)

PVs displayed nucleotide and amino acid tree lengths globally homogeneous across the individual genes (Fig. 5a and b). There were no significant trends in tree length with gene order neither for nucleotide (*R*^2^ = 0.26; *F*_1,4_ = 1.376, *p* = 0.31) nor for amino acid (*R*^2^ = 0.39; *F*_1,4_ = 2.57, *p* = 0.18). Grouping the tree lengths using a *K*-means cluster and choosing the number of clusters after the AIC, the best clustering at the nucleotide level was with two clusters: E6 and E7 in one and the remaining genes in the other. At the amino acid level, the lowest and indistinguishable AIC values were for 2 (E6-E7-E2 and L1-L2-E1) and three clusters (E7, E6-E2-L2 and E1-L1).

The first four columns of Additional file 5: Table S3b show the projection values in the first two dimensions of the PCoA of the pairwise distances between the individual gene trees, considering a combination of topology and branch length (*K*-score) (Fig. 5c and d) [56]. In both PCoA, the first axis spreads E6 (positive value), E7 (negative value) and a group formed by all other genes (values close to 0).

Regarding selection regime, PV genes presented a central estimator for *ω* ranging from 0.08 to 0.6 (Fig. 5e), with E7 gene showing the highest *ω* = 0.60 ± 0.37. Also, 25 % of the E7 codons were under neutral or positive selection, while all codons in other genes are under purifying selection, except 1.8 % of positions under neutral or positive selection in E2.

**Fig. 6:**
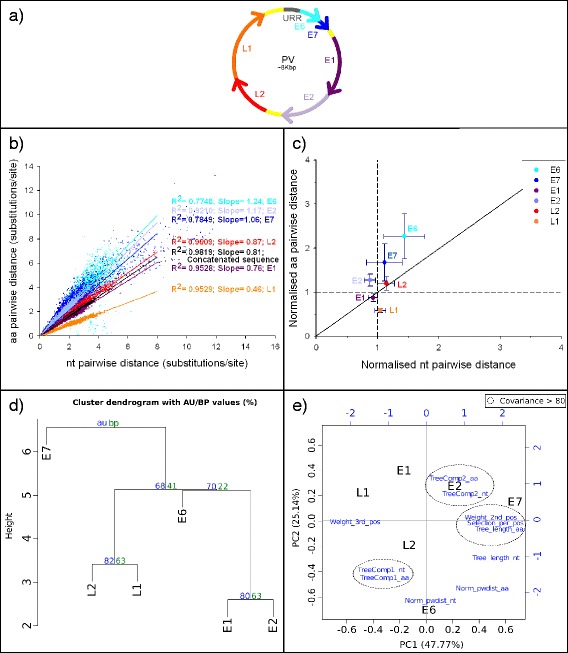
**a** PV genome organisation. **b** Nucleotide distance *vs* amino acid distance for PVs. Slopes values for each regression are indicated. All regression *p* < 0.001. **c** Nucleotide *vs* amino acid pairwise distances for PV genes. For each gene, the Huber estimator (±median absolute deviation) of the distances normalised to their respective concatenated is represented. **d** Hierarchical cluster dendogram using the eleven chosen variables (see Additional file 5: Table S3b) for PV. The clustering was performed using the euclidean distances and the Ward method. Probability values were calculated using bootstrap resampling techniques, the approximately unbiased (AU) support *p*-value (red) and the bootstrap probability (BP) value (red). **e** Biplot of the principal component analysis (PCA) using the eleven chosen variables (see Additional file 5: Table S3b) for PV. The first principal component is represented in the x-axis, and the second principal component is represented in the y-axis. Percentage values in the axes show the percentage of variation explained by each components. Original variables are given in blue, and those showing co-variation above 0.8 are encircled by discontinuous lines

The organisation of the genes into the PV genome is shown in Fig. 6a. Plotting nucleotide *pwdist* against amino acid *pwdist* yielded always a high correlation, for each individual gene as well as for the concatenated, but the slope of the regression varied largely depending on the gene (see example in Fig. 6b). Plotting *Norm_pwdist_nt vs Norm_pwdist_aa* showed that E6 was the most divergent gene with respect to the complete genome, displaying the highest distances at both the amino acid and the nucleotide levels (Fig. 6c). In contrast, E1 and L1 were more conserved than the complete genome at the amino acid level, and E1 and E2 were more conserved than the complete genome at the nucleotide level.

The cluster analysis of the eleven variables extracted for the six analysed genes (Additional file 5: Table S3) is given in Fig. 6d. The cluster analysis showed a clear separation of E7 from the rest of genes. Then genes involved in building the virus capsid, L2 and L1, clustered with an AU support *p* = 0.85 and early genes involved in genome replication, E1 and E2, clustered together with an AU support *p* = 0.82. Regarding the PCA, the eleven observed variables could be rescaled into two principal components that explained 72.74 % of the total observed variation (Fig. 6e). Regarding the genes, the first component discriminated E7 from the other genes and the second component separated E6 from the other genes. Covariance values above 80 clustered the eleven considered variables into three groups and four isolated variables.

## Discussion

We describe here an integrative approach to identify groups of genes sharing common patterns of evolution in genomes with reduced size, and exemplified the validity of the method by applying it to two viral genome datasets, differing largely in genome structure as well as in evolutionary distance among the taxa included. We chose two data sets with quite different characteristics to illustrate both the applicability of the method in distinct situations and how the outcome can be used and interpreted depending on these situations. The TuMV data set is an example of RNA viruses with low divergence between the terminal leaves and a moderate knowledge of the biology of the virus whereas the PVs data set is an example of DNA viruses with high divergence between the terminal leaves and a good knowledge of the viruses biology.

The method here depicted combines a series of eleven variables recapitulating information about tree topology and branch length, about agreement between gene-based and genome-based phylogenetic reconstructions, about the strength of selection along the positions in a gene, and about comparison between gene-based and genome-based distances between taxa. These eleven variables are not fully independent from one another, but covariance patterns between variables are different for the two viral datasets, thus showing that none of them can a priori be considered as redundant. The result provides with visual, intuitive plots easy to interpret, which can guide further informed comparisons, when incorporating knowledge on gene function. Thus, interpreting nucleotide *vs* amino acid pairwise distances with the key provided in the graphical representation of the normalised amino acid distance *vs* the normalised nucleotide distance shows distinct evolutionary patterns for both datasets, and allows pinpointing highly divergent genes, e.g. P1 in TuMV genomes and E6 and E7 in PVs. Further, we have applied information reduction techniques that allow projecting the 11-dimension space into more visual two-dimensional plots. Again, obvious trends of similarity in evolutionary patterns are evidenced and can be tracked back to biological differences between genes, as in the split between the E6 and E7 genes and the rest of the PV genes, mirroring the proposed evolutionary history for the blocks composing the PV genomes [66, 67].

For TuMV genes, we identified two major gene clusters grouping together genes that are physically close in the viral genome. Certain genes are well conserved among isolates (NIb, CP) while others are more divaricated (P1, P3). Our results show that P1 presents a very distinct evolutionary pattern, in terms of increased positive selection, long amino acid tree length and large pairwise amino acid distances. P1 is known to be diverse in sequence and length both within and between species [68–70]. It has also been established that intragene recombination and gene duplication contributed to the evolution of P1 and to successful host adaptation [45]. Moreover, the non-proteolytic part, i.e. a very large portion of the protein, is dispensable for infectivity and replication in *Tobacco etch potyvirus* (TEV) [71], suggesting a lower level of selective constraint on this protein. Finally, P1 plays a role in determining the virus host range [72, 73]. This role implies that the P1 protein carries the hallmark of either a process of adaptation to the host or of an evolutionary arms race with the host(s). These two phenomena are likely responsible for the observed pattern of positive selection and increased amino acid diversity, which cluster P1 apart from the rest of the TuMV genes.

One striking pattern for TuMV is that the final clustering closely follows the gene order in the genome. Gene order is known to be very essential for potyvirus functioning as recently demonstrated experimentally in TEV [74]: the NIb gene, encoding for the polymerase, was relocated at all possible intercistronic positions and all relocations were lethal to the virus except when NIb was placed before P1 or between P1 and HC-Pro. This experimental result also adds to the idea that the 5’ side of the potyvirus genome is more permissive to changes. As for the origin of the relationship between gene order and evolutionary history outlined by our study, it could go both ways: gene order could drive evolutionary history or evolutionary history could drive gene order. In the first, case, we have here a higher degree of conservation for the 3’ proteins that could be due to an increase in the selection pressure along the genome. However, we cannot totally exclude a decrease in the error rate of the NIb RNA-dependent-RNA polymerase along the genome, even though it has never been documented. Regarding the potential increase in selection pressure along the genome, it is important to keep in mind that the genome itself serves as mRNA and is translated into a polyprotein, which is then cleaved in ten functional proteins. Hitherto, no mechanisms for differential regulation of individual protein expression have been described, meaning that all proteins are expected to be synthesised simultaneously and in similar amounts. However, such uniformity for protein products with different functions and requirements is unlikely. A recent study on the P1 protease of *Plum pox potyvirus,* reveals a modulation of its activity. This P1 protease activity regulation could allow for a fine modulation on the viral amplification and reduce triggering of host immune responses [75]. This recent study is a first element arguing for differences in expression levels, potentially causing differences in selection intensity. An alternative mechanism for the regulation of the expression of individual proteins would be the presence of internal ribosome entry segment (IRES). This mechanism has been identified in picornaviruses [76] and in the *Shrimp white spot syndrome virus* [77]. The same phenomenon could apply to the single TuMV ORF with a higher expression of 3’ proteins than of 5’ proteins, leading to a more stringent selection on 3’ proteins.

In the context of the reverse causality – evolutionary history drives gene order - the observed clustering could reflect the organisation of the genome in groups of proteins that interact together for the realisation of the same function: host-virus interaction (P1), accessory factors of genome replication (HC-Pro, P3, CI) and core replicase (6 K2, VPg, NIb). An evolutionary advantage of such genome organisation would be that interacting proteins are released simultaneously. However the “functional group” argument is not very appropriate for compact viral genomes with multifunctional proteins involved in multiple interactions. For example, CI and CP have been described as having a role in virus cell-to-cell movement [78–80] but do not cluster in terms of evolutionary patterns.

Regarding PVs, the results are consistent with the gene expression patterns and with the natural history of the viral infection. Both clustering and pairwise distances analyses reveal that PV genes are organised in two main blocks: the first one composed by genes involved in the replication of the viral genome, and the second one composed by genes involved in the encapsidation of the virus. These two blocks are accompanied by the oncogenes E6 and E7, which are not grouped in any cluster. This result is consistent with the hypothesis suggesting that the proto-PV was composed by the E1, E2, L2 and L1 genes, the core region of the genome, while the E6 and E7 were incorporated later, providing with dispensable transforming capacities [66]. Further, this clustering matches well differences in codon usage preferences between different PV genes, which are similar for genes expressed at similar stages of the natural history of the PV infection [81].

The two blocks of the PV genome encompasses the only four genes that are present in all PVs and that may potentially suffice for completing the viral infection cycle [66]. They encode for the L1 and L2 proteins, which form the viral capsid [82]; for the E1 protein, which binds DNA, recruits cellular factors for DNA replication and acts as a helicase [83–85]; and for the E2 protein, a transcription factor that modulates viral gene expression and also directs E1 activity [86, 87]. The L1 gene is under a strong purifying selection, likely reflecting the essentially structural role of the L1 proteins, which are able to spontaneously self-assemble into virions [88]. The E1 and the L2 genes show similar global ratios of synonymous and non-synonymous mutations, as both lie on the reference diagonal for the concatenated genome, although the L2 gene accumulates more changes. Finally, the E2 gene contains a small number of positions identified to be under positive selection. These positions may indeed be under selection or may instead reflect the particular architecture of this protein: the N- and C-termini are well-conserved and interact forming an internal dimer to bind DNA [87], while the central, hinge region is poorly conserved and consists of stretches rich in proline, serine and glycine [89–93]. The filtering step for the sequence alignment previous to phylogenetic inference identifies most of the E2 hinge region to be poorly conserved and consequently removes it. The few positions under positive selection identified in the E2 gene map to the remnants of the hinge region that have been selected for tree construction. The hinge region of the E2 gene accommodates the E4 ORF overlapping in a ±1 frame. In this hinge region, the pressure towards conservation of the E4 amino acid positions renders synonymous changes in the E4 frame as non-synonymous changes in the E2 frame [94]. This E4 gene has not been included in our analyses because it is properly annotated only in a few genera of the *Papillomaviridae* family.

The isolated genes E6 and E7 are not present in all PVs, as some PVs encode E7 but no E6 proteins [95–97] whereas other PVs encode E6 but no E7 proteins [46, 98, 99]. For the best studied PVs, the role of these proteins during PV infection is the disruption of the growth host cell control by interacting with the tumour suppressor proteins p53 and pRb in the upper layers of the epithelium [100–102]. The expression of both genes drives duplication of keratinocytes in skin layers in which no replication normally occurs, and prevents checkpoint mechanisms from triggering cell arrest [103–105]. Both E6 and E7 proteins are small and highly disordered and have multiple interaction partners [106–109]. These structural features, together with the fact of being dispensable in the PV genome, constitute the main differences between the E6 and E7 proteins and those present in the PV core genome, E1-E2-L2-L1. The results in our analyses for PVs deepen those from previous reports [65, 66, 81] and reflect also these fundamental differences, gathering the inconsistency in phylogenetic relationships inferred for the two oncogenes, the increase in accepted nucleotide substitutions, and the large fraction of positions under positive selection for E7, and leading to the clear split of the PV genes into two clusters that globally reflect PV biology and evolution.

## Conclusion

The idea that viral genes might have different evolutionary histories is not new, particularly for virus with segmented genomes (e.g. [110, 111]). However, comparisons are often qualitative and use only part of the information that can be extracted from the sequences. The comprehensive analysis presented here allows identifying characteristics of the evolution of individual genes and to pinpoint groups of genes with similar evolutionary patterns in terms of phylogenetic relationships and evolutionary pressures. The two data sets we used illustrate that this strategy can be applied to different evolutionary scales: the TuMV data set gathered sequences of variants of a virus species with a divergence of 10^-2^ substitutions/position/taxon, whereas the PV data set was constituted of sequences of species within the *Papillomaviridae* family with 10^-1^ substitutions/position/taxon. This difference of evolutionary scale is actually reflected in the 20 times difference in tree length. The viral genomes in the example data sets were of the same length range (10 kb), but the method could be applied to larger viruses or to bacterial genomes. For larger genomes the procedure described here could allow identifying gene clusters with similar evolutionary pattern within the core genome of bacteria, as it has allowed us in the case of the PVs to infer the evolutionary steps prior to the appearance of modern PV genomes.

With this first attempt to combine evolutionary and phylogenetic information on the orthologous genes of sets of isolates, we have shown, in an integrative way, that inconsistencies between gene trees can be exploited to identify groups of genes with similar evolutionary histories. By choosing two viral data sets with very distinct characteristics, we illustrated that this method (1) can be applied to very compact genomes, (2) is able to recover from an evolutionary point of view the functional data accumulated on well-characterized virus or (3) to unravel unknown characteristics of the evolutionary history, likely related to protein functions, of less studied viruses. This opens perspectives for the generation of evolutionary and functional hypotheses on the basis of sequence data in general and for a refinement of core-genome determination in particular.

## Additional files

Additional file 1: **Supplementary material and methods.** Exhaustive workflow description indicating the input and output files for each step and where to find the software resources. This workflow combined with Fig. 1 allows full reproduction of the method. (PDF 1916 kb)

Additional file 2: Table S1. Accession numbers of TuMV (a) and PV (b) genomes used to perform the analysis.

Additional file 3: Figure S1. Best-known ML tree (a) and phylogenetic network (d) constructed from the TuMV concatenated nucleotide data set. Around them, the best-known ML tree constructed for each of the genes of TuMV at nucleotide level. Shaded areas correspond to the supported groups refered to in the text and in Additional file 4: Table S2a.

Additional file 4: Table S2. Bootstrap support for the clusters identified (see Additional file 3: Figure S1 and Additional file 6: Figure S2) in the phylogenetic trees built from the concatenated sequence for the concatenated tree and the gene trees, for the TuMV and the PV nucleotide data set.

Additional file 5: Table S3. Values of the eleven variables extracted from different characteristics of the evolution of each of the genes, such as tree-topology, branch length, detection of the level of selection operating on the proteins and phylogenetic distances between taxa.

Additional file 6: Figure S2. Best-known ML tree (a) and phylogenetic network (d) constructed from the PV concatenated nucleotide data set. Around them, the best-known ML tree constructed for each of the genes of PV at nucleotide level. Shaded areas correspond to the supported groups refered to in the text and in Additional file 4: Table S2b.
