# Supplementary material for: Assessing parallel gene histories in viral genomes
Source: BMC Evol Biol. 2016 Feb 5;16:32. doi: 10.1186/s12862-016-0605-4 (PMC4743424; doi:10.1186/s12862-016-0605-4)
Supplement: Supplementary file 1 — Supplementary material and methods. Exhaustive workflow description indicating the input and output files for each step and where to find the software resources. This workflow combined with Fig. 1 allows full reproduction of the method. (PDF 1916 kb) [file 12862_2016_605_MOESM1_ESM.pdf]

Supplementary material and methods

Perform the alignment:

1. Download, both, nucleotides –*gene_seqs_nt.fas* (1)– and amino acid –*gene_seqs_aa.fas* (2)– gene sequences from the databases. Save the sequences of each of the genes in separated files.

   Example:

   E1gene_seq_nt.fas file only contains the gene E1 from the different organisms

   > Organism 1

   atggctgaaactgcaggtagctcggggcagggggggggagcttatatctgctttgaagccgactgtagcgactctgatacagaggtt…..

   > Organism 2

   atggacgacggtaaaccaggtactggacagtattctggatggttttatattgacagggaggctgaatgtaatgataatgatgtggagga…..

   > Organism 3

   atggcaaatgagaaaggtagcaattgggattcgggcttgggatgctcatatctgctgactgaggcagaatgcgaaagtgacaaagat…..

2. Perform the alignment for each of the amino acid files, with the MUSCLE software (online version: http://www.ebi.ac.uk/Tools/msa/muscle/; version for download: http://www.drive5.com/muscle/downloads.htm).

   Input file: *gene_seqs_aa.fas* (1)

   Output file: *gene_seqs_alin_aa.fas* (3)

3. Perform the corresponding nucleotide alignments with the on-line software PAL2NAL (http://www.bork.embl.de/pal2nal/).

   Input files: *gene_seqs_alin_aa.fas* (3)
   gene_seqs_nt.fas (2)

   Output file: *gene_seqs_alin_nt.fas* (4)

4. Alignment pruning with Gblocks (version for download: http://molevol.cmima.csic.es/castresana/Gblocks.html).

   Input file: *gene_seqs_alin_nt.fas* (4)

   Output file: *gene_seqs_alin_gb_nt.fas* (5)

   Parameters:

   - Minimum number of sequences for a conserved position: half of the sequences plus one.
   - Minimum number of sequences for a flank position: the same number as the previous parameter.
   - Maximum number of contiguous non-conserved positions: 12.
   - Minimum length of a block: 6

- Allowed gap positions: all.

5. Gene concatenating with BioEdit software (version for download: http://www.mbio.ncsu.edu/bioedit/bioedit.html).

   Input files: All *gene_seqs_alin_gb_nt.fas* (5) files

   Output file: *concatenate_seqs_alin_gb_nt.fas* (6)

6. Translating nucleotide files into amino acid files with the Mega software (version for download: http://www.megasoftware.net/).

   Input files: All *gene_seqs_alin_gb_nt.fas* (5) files
   concatenate_seqs_alin_gb_nt.fas (6)

   Output files: All *gene_seqs_alin_gb_aa.fas* (7) files
   concatenate_seqs_alin_gb_aa.fas (8)

Phylogenetic reconstruction:

1. Maximum likelihood phylogenetic reconstruction with RAxML software (version for download: http://sco.h-its.org/exelixis/web/software/raxml/index.html).

   Input files: All *gene_seqs_alin_gb_nt.fas* (5) files
   concatenate_seqs_alin_gb_nt.fas (6)
   All gene_seqs_alin_gb_aa.fas (7) files
   concatenate_seqs_alin_gb_aa.fas (8)

   Output files: All *Best_tree_gene_seqs_alin_gb_nt.fas* (9) files
   All *Best_tree_info_gene_seqs_alin_gb_nt.fas* (10) files
   Best_tree_concatenate_seqs_alin_gb_nt.fas (11)
   Best_tree_info_concatenate_seqs_alin_gb_nt.fas (12)
   All *Best_tree_gene_seqs_alin_gb_aa.fas* (13) files
   All *Best_tree_info_gene_seqs_alin_gb_aa.fas* (14) files
   Best_tree_concatenate_seqs_alin_gb_aa.fas (15)
   Best_tree_info_concatenate_seqs_alin_gb_aa.fas (16)

   Parameters:

   - raxmlHPC -f a -s input_file -x 123 -# autoMRE -m substitution_model -d -n output_file
     (Note: to obtain the variables Weight_2nd_pos and Weight_3rd_pos assign distinct models to the codon positions, create the partition_file with the following contents:
     DNA, gene1codon1 = 1-500\3
     DNA, gene1codon2 = 2-500\3
     DNA, gene1codon3 = 3-500\3
     and please add "-q partition_file -M" in the command line).

The variables Tree_length_nt (mean of the partition Tree-Lengths), Tree_length_aa (mean of the partition Tree-Lengths), Weight_2nd_pos (ratio between Tree-Length of the 1st partition of the nucleotide trees and Tree_length_nt), and Weight_3rd_pos (ratio between Tree-Length of the 2nd partition of the nucleotide trees and Tree_length_nt) will be obtained from the files all *Best_tree_info_gene_seqs_alin_gb_nt.fas* (10) files, and all *Best_tree_info_gene_seqs_alin_gb_aa.fas* (14) files.

Phylogeny tree comparison:

1. Distances comparison by K-score implemented in the Ktreedist software (version for download: http://molevol.cmima.csic.es/castresana/Ktreedist.html)

   Input files: All *Best_tree_gene_seqs_alin_gb_nt.fas* (9) files
   All *Best_tree_gene_seqs_alin_gb_aa.fas* (13) files

   Output: The *K*_score_nt and *K*_score_aa values.

2. Processing the *K*-score values using the Principal Coordinate Analysis (PCoA) implemented in the "vegan" and "ade4" R Packages (version for download: http://www.r-project.org/)

   Input: A matrix with the *K*-score values. A matrix for nucleotides and another one for amino acids.

   Output: The values of the first two dimensions of the PCoA will be considered the variables TreeComp1_nt, TreeComp2_nt, TreeComp1_aa and TreeComp2_aa.

Identification of Selective pressures:

1. Site-specific positive and purifying selection is measured with the Selecton web server (online version: http://selecton.tau.ac.il/).

   Input files: All *Best_tree_gene_seqs_alin_gb_dist_nt.fas* (17) files
   All *gene_seqs_alin_gb_nt.fas* (5) files

   Output: The $\omega = d_N/d_S$ ratio at the individual codon level

2. Measuring the central tendency of the ω through the Huber M-estimator, implemented in the "MASS" R Packages (version for download: http://www.r-project.org/).

   Input: The $\omega = d_N/d_S$ ratio at the individual codon level

   Output: The variable Selection_per_pos at the gene level

Tree distances calculation:

1. Calculation of nucleotide and amino acid distances for all possible pairs for each gene and the concatenate sequence. It is performed through the RAxML software (version for download: http://sco.h-its.org/exelixis/web/software/raxml/index.html).

   Input files: All *gene_seqs_alin_gb_nt.fas* (5) files
   concatenate_seqs_alin_gb_nt.fas (6)
   All gene_seqs_alin_gb_aa.fas (7) files
   concatenate_seqs_alin_gb_aa.fas (8)
   All *Best_tree_gene_seqs_alin_gb_nt.fas* (9) files
   Best_tree_concatenate_seqs_alin_gb_nt.fas (11)
   All *Best_tree_gene_seqs_alin_gb_aa.fas* (13) files
   Best_tree_concatenate_seqs_alin_gb_aa.fas (15)

   Output files: All *Best_tree_gene_seqs_alin_gb_dist_nt.fas* (17) files
   Best_tree_concatenate_seqs_alin_gb_dist_nt.fas (18)
   All *Best_tree_gene_seqs_alin_gb_dist_aa.fas* (19) files
   Best_tree_concatenate_seqs_alin_gb_dist_aa.fas (20)

   Parameters:
   - raxmlHPC -f x -s alignment_input_file -t Best_tree_input_file -m substitution_model -n output_file
     (Note: please add "-q partition_file" in the command line when it is necessary)

3. Normalising the nucleotide and amino acid distances respect to their corresponding concatenated. Subsequently, measuring the Huber M-estimator, implemented in the "MASS" R Packages (version for download: http://www.r-project.org/).

   Input files: All *Best_tree_gene_seqs_alin_gb_dist_nt.fas* (17) files
   Best_tree_concatenate_seqs_alin_gb_dist_nt.fas (18)
   All *Best_tree_gene_seqs_alin_gb_dist_aa.fas* (19) files
   Best_tree_concatenate_seqs_alin_gb_dist_aa.fas (20)

   Output: The variables Norm_pwdist_nt and Norm_pwdist_aa

Identifying genes displaying similar evolutionary metrics:

Use de following variables: Tree_length_nt, Tree_length_aa, Weight_2nd_pos, Weight_3rd_pos TreeComp1_nt, TreeComp2_nt, TreeComp1_aa, TreeComp2_aa, Selection_per_pos Norm_pwdist_nt and Norm_pwdist_aa.

1. Standardise the variables and perform a Ward hierarchical clustering using the Euclidean distances through the R package "pvclust" (version for download: http://www.r-project.org/).

2. Use these same standardised variables to perform a principal component analysis (PCA) through the R package "stats" (version for download: http://www.r-project.org/).
