# Supplementary material for: Assessing parallel gene histories in viral genomes
Source: BMC Evol Biol. 2016 Feb 5;16:32. doi: 10.1186/s12862-016-0605-4 (PMC4743424; doi:10.1186/s12862-016-0605-4)
Supplement: Supplementary file 2 — Accession numbers of TuMV (a) and PV (b) genomes used to perform the analysis. [file 12862_2016_605_MOESM2_ESM.pdf]

Table S1a

TuMV name	Name of the first host from which the TuMV was isolated	GenBank ID
2J	*Brassica pekinensis*	AB093622
59J	*Raphanus sativus*	AB093620
A102/11	*Anemone coronaria*	AB093597
A64	*Anemone coronaria*	AB093599
Al	*Alliaria officinalis*	AB093598
BZ1	*Brassica oleracea*	AB093611
C42J	*Brassica rapa*	AB093625
Cal1	*Calendula officinalis*	AB093601
CDN1	*Brassica napus*	AB093610
CHN12	Not known	AY090660
CP845J	*Calendula officinalis*	AB093614
CZE1	*Brassica oleracea*	AB093608
DMJ	*Raphanus sativus*	AB093623
HRD	*Raphanus sativus*	AB093627
IS1	*Allium ampeloprasum*	AB093602
ITA7	*Raphanus raphanistrum*	AB093600
Ka1J	*Brassica pekinensis*	AB093624
KD32J	*Raphanus sativus*	AB093621
KEN1	*Brassica oleracea*	AB093605
KYD81J	*Raphanus sativus*	AB093613
NZ290	*Brassica pekinensis*	AB093612
PV0104	*Lactuca sativa*	AB093603
PV376-Br	*Brassica napus*	AB093604
Q-Ca	*Brassica rapa*	D10927
RUS1	*Armoracia rusticana*	AB093606
RUS2	*Brassica napus*	AB093607
St48	*Limonium sinuatum*	AB093596
TW	Not known	AF394602
UK1	*Brassica napus*	AF169561
USA1	*Brassica oleracea*	AB093609

Table S1b

PV name	Name of the first host from which the PV was isolated	GenBank ID
AaPV1	*Alces alces*	M15953
BPV1	*Bos taurus*	X02346
BPV7	*Bos taurus*	DQ217793
BPV8	*Bos taurus*	DQ098913
CcaPV1	*Capreolus capreolus*	EF680235
CcPV1	*Caretta caretta*	EU493092
ChPV1	*Capra hircus*	DQ091200
CmPV1	*Chelonia mydas*	EU493091
CPV1	*Canis familiaris*	D55633
CPV2	*Canis familiaris*	AY722648
CPV4	*Canis familiaris*	EF584537
CPV5	*Canis familiaris*	FJ492743
CPV6	*Canis familiaris*	FJ492744
CPV7	*Canis familiaris*	FJ492742
EcPV1	*Equus caballus*	AF498323
EcPV2	*Equus caballus*	EU503122
EdPV1	*Erethizon dorsatum*	AY684126
EePV1	*Erinaceus europaeus*	FJ379293
FcaPV1	*Felis domesticus*	AF480454
FcaPV2	*Felis domesticus*	EU796884
HPV6	*Homo sapiens*	X00203
HPV10	*Homo sapiens*	X74465
HPV13	*Homo sapiens*	X62843
HPV16_C	*Homo sapiens*	AF472509
HPV18	*Homo sapiens*	X05015
HPV24	*Homo sapiens*	U31782
HPV26	*Homo sapiens*	X74472
HPV31	*Homo sapiens*	J04353
HPV32	*Homo sapiens*	X74475
HPV36	*Homo sapiens*	U31785
HPV38	*Homo sapiens*	U31787
HPV39	*Homo sapiens*	M62849
HPV40	*Homo sapiens*	X74478
HPV41	*Homo sapiens*	X56147
HPV44	*Homo sapiens*	U31788
HPV45	*Homo sapiens*	X74479
HPV50	*Homo sapiens*	U31790
HPV54	*Homo sapiens*	AF436129
HPV56	*Homo sapiens*	X74483
HPV57	*Homo sapiens*	X55965
HPV58	*Homo sapiens*	D90400
HPV60	*Homo sapiens*	U31792
HPV61	*Homo sapiens*	U31793
HPV63	*Homo sapiens*	X70828
HPV65	*Homo sapiens*	X70829
HPV73	*Homo sapiens*	X94165
HPV76	*Homo sapiens*	Y15174
HPV80	*Homo sapiens*	Y15176
HPV85	*Homo sapiens*	AF131950
HPV87	*Homo sapiens*	AJ400628
HPV90	*Homo sapiens*	AY057438
HPV92	*Homo sapiens*	AF531420
HPV96	*Homo sapiens*	AY382779
HPV102	*Homo sapiens*	DQ080083
HPV104	*Homo sapiens*	FM955840
HPV109	*Homo sapiens*	EU541441
LrPV1	*Lynx rufus*	AY904722
MaPV1	*Mesocricetus auratus*	E15111
McPV2	*Mastomys coucha*	DQ664501
MfPV1	*Macaca fascicularis*	EF028290
MfPV3_b	*Macaca fascicularis*	EF591299
MfPV6	*Macaca fascicularis*	EF558840
MfPV8	*Macaca fascicularis*	EF558842
MmiPV1	*Micromys minutus*	DQ269468
MnPV1	*Mastomys natalensis*	U01834
OaPV1	*Ovis aries*	U83594
OcPV1	*Oryctolagus cuniculus*	AF227240
OvPV1	*Odocoileus virginianus*	M11910
PcPV1	*Puma concolor*	AY904723
PlpPV1	*Panthera leo persica*	AY904724
PlPV1	*Procyon lotor*	AY763115
PpPV1	*Pan paniscus*	X62844
PtPV1	*Pan troglodytes*	AF020905
RaPV1	*Rousettus aegyptiacus*	DQ366842
RnPV1	*Rattus norvegicus*	GQ180114
RtPV1	*Rangifer tarandus*	AF443292
SfPV1	*Sylvilagus floridanus*	K02708
TmPV1	*Trichechus manatus latirostris*	AY609301
UuPV1	*Uncia uncia*	DQ180494
