# Supplementary figures and images for: Assessing parallel gene histories in viral genomes
Source: BMC Evol Biol. 2016 Feb 5;16:32. doi: 10.1186/s12862-016-0605-4 (PMC4743424; doi:10.1186/s12862-016-0605-4)

Figure S1

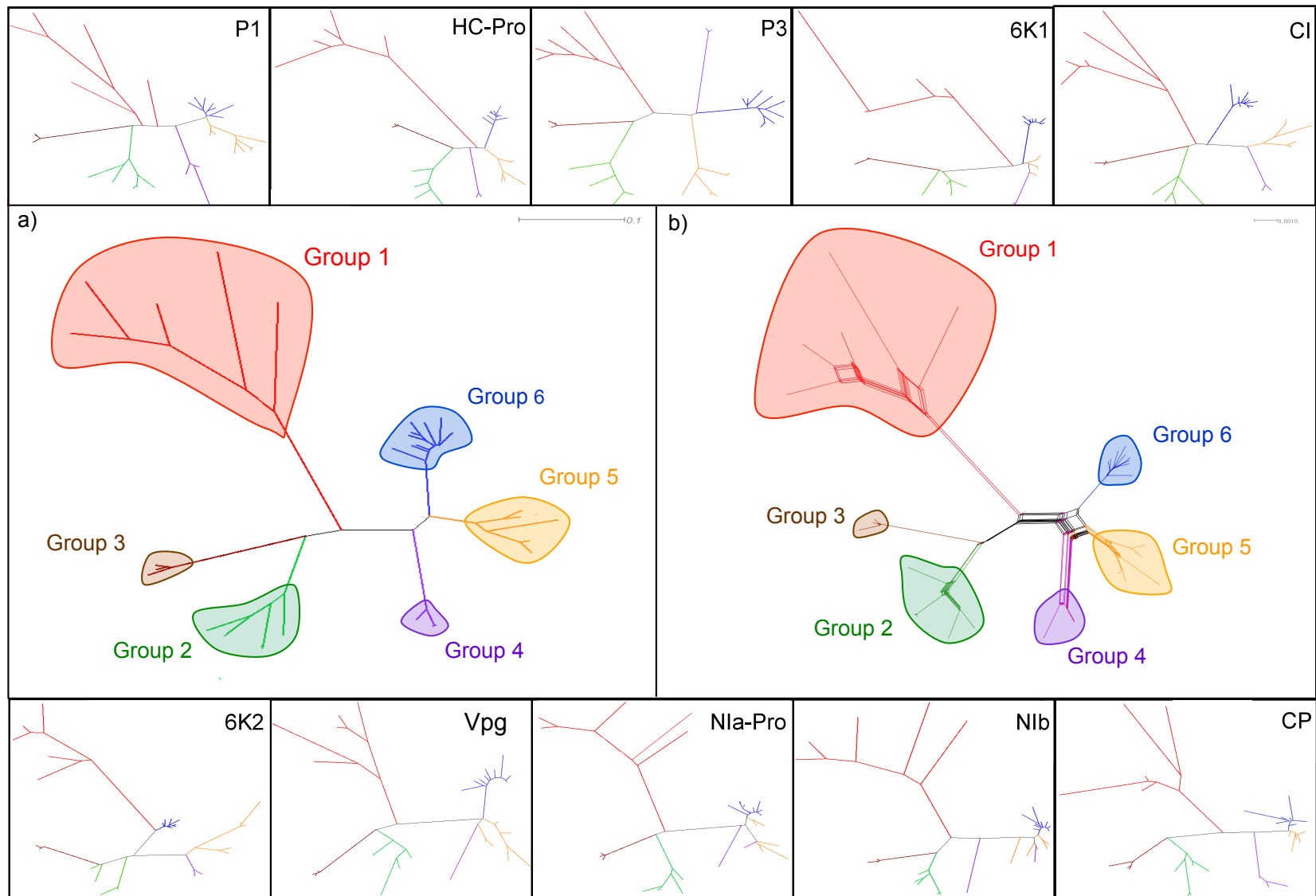

Supplement: Supplementary file 3 — Best-known ML tree (a) and phylogenetic network (d) constructed from the TuMV concatenated nucleotide data set. Around them, the best-known ML tree constructed for each of the genes of TuMV at nucleotide level. Shaded areas correspond to the supported groups refered to in the text and in Additional file 4: Table S2a. [file 12862_2016_605_MOESM3_ESM.pdf]

Figure S2

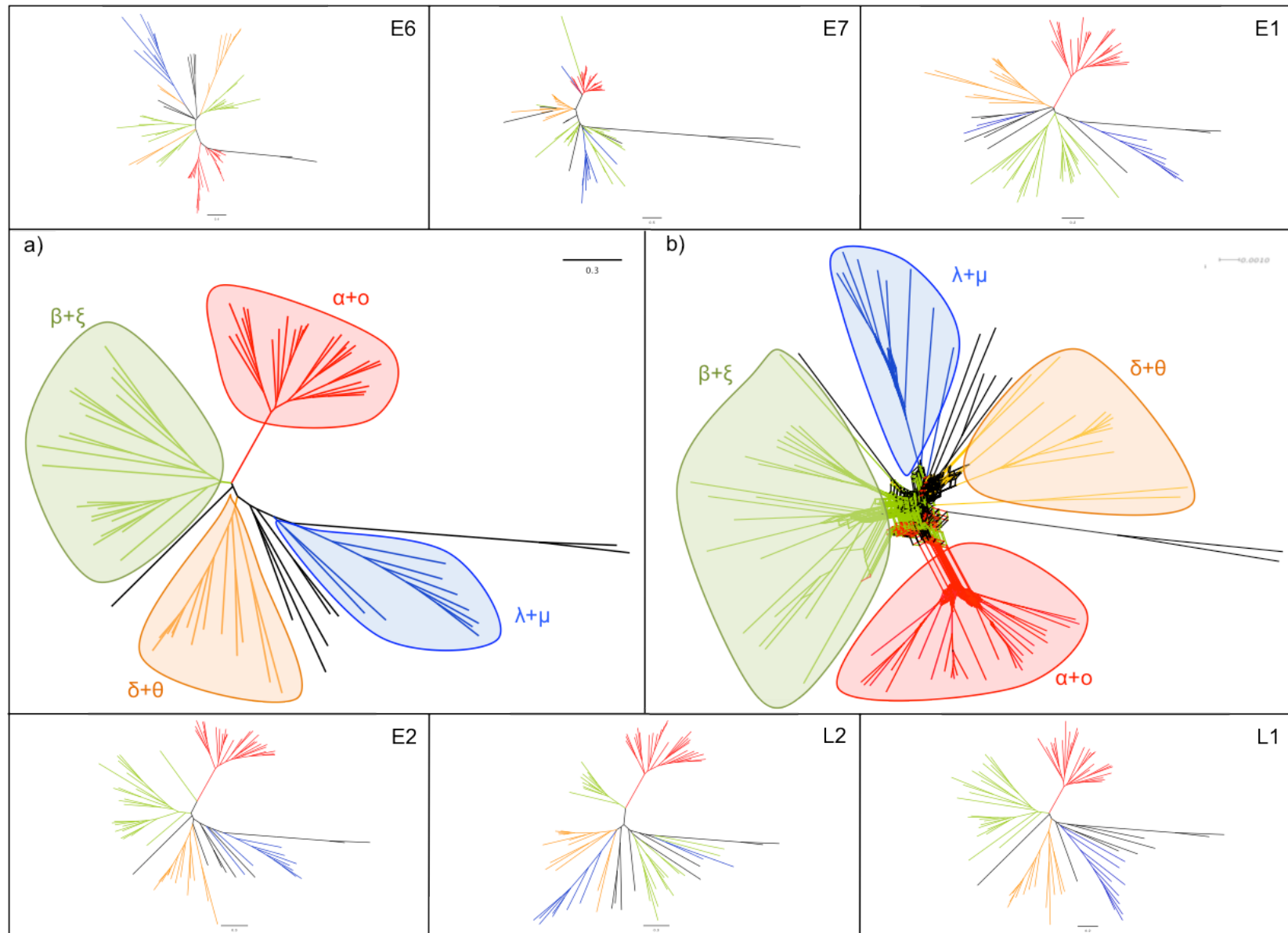

Supplement: Supplementary file 6 — Best-known ML tree (a) and phylogenetic network (d) constructed from the PV concatenated nucleotide data set. Around them, the best-known ML tree constructed for each of the genes of PV at nucleotide level. Shaded areas correspond to the supported groups refered to in the text and in Additional file 4: Table S2b. [file 12862_2016_605_MOESM6_ESM.pdf]
