# Supplementary material for: Assessing parallel gene histories in viral genomes
Source: BMC Evol Biol. 2016 Feb 5;16:32. doi: 10.1186/s12862-016-0605-4 (PMC4743424; doi:10.1186/s12862-016-0605-4)
Supplement: Supplementary file 4 — Bootstrap support for the clusters identified (see Additional file 3: Figure S1 and Additional file 6: Figure S2) in the phylogenetic trees built from the concatenated sequence for the concatenated tree and the gene trees, for the TuMV and the PV nucleotide data set. [file 12862_2016_605_MOESM4_ESM.pdf]

Table S2

a)

TuMV	Group 1	Group 2	Group 3	Group 4	Group 5	Group 6
P1	92(except St48_Italy)	100	100	100	88	-
HC-Pro	100	100	100	100	99	100
P3	100	100	100	100	100	98
6K1	82	79(except ITA7_Italy)	96	78	-	87
CI	100	100	100	100	86	100
6K2	100	-	99	78	86	74
VPg	100	88	100	-	-	98
NIa-Pro	100	100	99	-	-	43
NIb	100	99	100	-	-	89
CP	100	78	97	100	53	53
Concatenated genes	100	100	100	100	92	100

b)

PV	α+o	β+ξ	λ+μ	δ+θ
E6	-	-	-	46
E7	-	-	-	60
E1	99	59	41	99
E2	100	18(except PBV7)	28	91
L2	100	-	66(except HPV41,EdPV)	100
L1	100	66	86	28
Concatenated genes	100	94	100	100
