# Supplementary material for: Assessing parallel gene histories in viral genomes
Source: BMC Evol Biol. 2016 Feb 5;16:32. doi: 10.1186/s12862-016-0605-4 (PMC4743424; doi:10.1186/s12862-016-0605-4)
Supplement: Supplementary file 5 — Values of the eleven variables extracted from different characteristics of the evolution of each of the genes, such as tree-topology, branch length, detection of the level of selection operating on the proteins and phylogenetic distances between taxa. [file 12862_2016_605_MOESM5_ESM.pdf]

Table S3

a)

	Tree_length_nt	Weight_2nd_pos	Weight_3rd_pos	Tree_length_aa	TreeComp1_nt	TreeComp2_nt	TreeComp1_aa	TreeComp2_aa	Selection_per_pos	Norm_pwdist_nt	Norm_pwdist_aa
**P1**	2.689	0.178	0.643	2.209	-0.141	0.013	0.297	0.045	0.287	1.121	2.988
**HC-Pro**	2.861	0.019	0.898	0.370	-0.202	-0.250	-0.014	-0.055	0.009	1.430	0.451
**P3**	2.518	0.082	0.772	1.305	-0.098	0.093	0.048	-0.156	0.044	1.431	2.356
**6K1**	2.825	0.002	0.955	0.161	-0.414	0.109	-0.103	0.041	0.008	1.355	0.386
**CI**	1.896	0.030	0.906	0.267	0.100	0.009	-0.006	0.031	0.008	0.881	0.388
**6K2**	2.040	0.050	0.810	0.761	0.137	-0.154	-0.069	-0.020	0.033	1.171	1.534
**VPg**	1.964	0.047	0.832	0.755	0.136	0.115	-0.041	0.010	0.018	0.991	1.246
**NIa-Pro**	1.902	0.008	0.903	0.181	0.192	0.014	-0.065	0.019	0.008	0.929	0.318
**NIb**	1.677	0.032	0.862	0.351	0.160	0.025	-0.016	0.055	0.017	0.732	0.435
**CP**	0.994	0.061	0.824	0.351	0.129	0.025	-0.032	0.029	0.025	0.463	0.504

b)

	Tree_length_nt	Weight_2nd_pos	Weight_3rd_pos	Tree_length_aa	TreeComp1_nt	TreeComp2_nt	TreeComp1_aa	TreeComp2_aa	Selection_per_pos	Norm_pwdist_nt	Norm_pwdist_aa
**E6**	41.669	0.145	0.617	39.400	1.958	0.245	2.405	0.025	0.249	1.434	2.267
**E7**	48.047	0.182	0.525	53.339	-2.311	0.163	-2.848	0.005	0.542	1.058	1.620
**E1**	30.867	0.135	0.645	31.396	0.030	0.408	0.048	0.535	0.167	0.926	0.874
**E2**	34.740	0.177	0.510	42.068	0.106	0.342	0.265	0.510	0.262	0.872	1.264
**L2**	38.391	0.143	0.633	37.141	0.151	-0.889	0.222	-0.975	0.169	1.136	1.194
**L1**	35.224	0.100	0.747	23.151	0.067	-0.270	-0.092	-0.099	0.068	1.044	0.589
